# Stock index trend prediction based on TabNet feature selection and long short-term memory

**DOI:** 10.1371/journal.pone.0269195

**Published:** 2022-12-13

**Authors:** Xiaolu Wei, Hongbing Ouyang, Muyan Liu

**Affiliations:** 1 Business School, Hubei University, Wuhan, Hubei, China; 2 Department of Economics, Huazhong University of Science and Technology, Wuhan, Hubei, China; 3 Business School, Sichuan University, Chengdu, Sichuan, China; TDTU: Ton Duc Thang University, VIET NAM

## Abstract

In this study, we propose a predictive model TabLSTM that combines machine learning methods such as TabNet and Long Short-Term Memory Neural Network (LSTM) with a complete factor library for stock index trend prediction. Our motivation is based on the notion that there are numerous interrelated factors in the stock market, and the factors that affect each stock are different. Therefore, a complete factor library and an efficient feature selection technique are necessary to predict stock index. In this paper, we first build a factor database that includes macro, micro and technical indicators. Successively, we calculate the factor importance through TabNet and rank them. Based on a prespecified threshold, the optimal factors set will include only the highest-ranked factors. Finally, using the optimal factors set as input information, LSTM is employed to predict the future trend of 4 stock indices. Empirical validation of the model shows that the combination of TabNet for factors selection and LSTM outperforms existing methods. Moreover, constructing a factor database is necessary for stock index prediction. The application of our method does not only show the feasibility to predict stock indices across different financial markets, yet it also provides an complete factor database and a comprehensive architecture for stock index trend prediction, which may provide some references for stock forecasting and quantitative investments.

## Introduction

The role of the stock indices is essential in the financial market since they reflect the macroeconomic health state and microeconomic issues of a particular market. Existing studies have suggested that despite the market is not efficient, stock indices can, to some extent, be characterized and predicted, allowing investors to identify and use arbitrage opportunities to make an excess profit [1–3]. The stock index is influenced by the economic cycle, market sentiments and corporate expectations, which increase the difficulty to elaborate suitable computational tools that capture the stock index dynamics [4]. Additional difficulties are also due to the intrinsic stochastic nature of the stock index, which shows non-linear, non-gaussian and non-stationary noise [5–7]. To address the abovementioned prediction challenges of a stock index, researchers have employed machine learning algorithms combined with features selection, which consistently outperform traditional econometric methods in terms of the predictive power of the stock index, while sacrificing their inferential aspects [8, 9].

In this paper, we present a novel deep learning model TabLSTM that not only allows researchers and practitioners in the financial field can employ to predict with low error rate the trend of a stock index, but it also extracts through a features selection procedure a set of factors that appear determinant for the characterization of the stock index. In particular, our modelling approach for predicting the stock index is composed of three stages: 1) Construction of a factors database at micro, macro and technical level; 2) Perform features selection procedure on the factor database by using TabNet. The selected features (optimal features set) determine the driving factors for predictive purposes; 3) Construction of a Long Short-Term Memory Neural Network (LSTM) model for stock index trend prediction.

To assess the performance of the model, four stock indices have been considered, namely S&P 500 index, Dow Jones Composite Index, China Shenzhen Composite Index and China Shanghai Composite Index. The features selection through TabNet is applied on each index and it leads to the identification of the four distinct optimal features sets, containing the likely important factor that characterizes the dynamics of the relative stock index. Finally, the optimal features set is used as input for the LSTM model, which predicts if the stock index will exhibit up- or down-trends. An out-of-sample evaluation of the model performances show evidence that our model outperforms other existing methods. Moreover, the construction of a comprehensive factor database is necessary for stock index prediction.

The contribution in this paper can be highlighted as follows:

- Build a comprehensive factor library from three perspectives: macro, micro and technical. This factor library includes both traditional factors such as price-to-earnings ratio (P/E) and emerging factors such as Google Attention Index.
- Study two distinct financial markets, including financial markets in developed countries and those in emerging countries.
- Use the original data without data preprocessing such as normalization, which ensure that important information is not lost.
- Study the current importance of each factor (local interpretability) and the impact of each factor on stock index trend (global interpretability) through TabNet method.

The rest of this paper is organized as follows. In section 2, fundamentals and concepts on market factors, features selection and trend prediction machine learning algorithms are discussed. In Section 3, details on TabNet and LSTM model and their application on stock index prediction are presented. In Section 4, data and evaluation metrics are described. In Section 5, experimental results of the proposed model are shown and discussed followed by a conclusion.

## Literature review

### 1. Information and price predictability of stock markets

The effectiveness of financial markets was first proposed by the French mathematician Bachelier [10] in the early 20th century. By analyzing the price changes in the stock market, he found that stock prices reflect almost all relevant information. Then in the 1960s, Fama et al. proposed the efficient market hypothesis (EMH) [11–13]. The efficient market hypothesis suggests that the stock prices at any given time should be a fair reflection of all currently available information, and that emerging information is quickly reflected in the stock prices. Therefore, analysis of historical and current data cannot help investors predict the future or earn excess profits. Depending on how prices respond to information, efficient market forms can be classified as weak-form efficient market, semi-strong-form efficient market and strong-form efficient market. The weak-form efficient market is consistent with the random wandering hypothesis, which states that stock prices fluctuate randomly and price changes are independent of each other. Moreover, the weak-form efficient market indicates that current stock price contains all available historical information and that it is impossible for investors to earn excess profits through technical analysis. The semi-strong-form efficient market assumes that stock prices adjust quickly to public information in market and that it is impossible to earn excess returns based on fundamental analysis. The strong-form efficient market assumes that stock prices reflect all available information in the market, including historical information, public information and relevant private information, so that no investor can earn excess profits by any means.

However, with further research, researchers have found that the two main assumptions of the efficient market hypothesis (no information costs, and "rational people" who seek to maximize their own interests) do not correspond to reality. At the same time, more and more anomalies in financial markets were discovered, and the efficient market hypothesis was unable to provide an effective and rational explanation for these anomalies.

In order to explain the anomalies in financial markets, Tversky and Kahneman (2013) first applied psychological assumptions to financial markets and proposed the "expectation theory" in 1979 [14]. In the theory, Tversky and Kahneman (1979) argued that investors have cognitive biases that conflict with the assumptions of the efficient market hypothesis due to risky attitudes, mental accounting and overconfidence. In 1989, Bhushan (1989) introduced the phenomenon of "herding effect" by analyzing the relationship between securities analysts and investors [15]. In 1993, Jegadeesh and Titman (1993) conducted an empirical study based on the average stock returns in the past six months and proposed the phenomenon of "return inertia" [16]. Latif et al. (2011) discussed different financial market anomalies and their causes [17]. Based on the empirical analysis, they suggested that investors could use calendar anomalies (weekend effect, monthly effect, annual effect and January effect), fundamental anomalies (value anomalies, low book price stocks, low yield price stocks, neglected stocks, high dividend yield stocks), technical analysis (moving averages) and insider trading to earn anomalous profits.

By reviewing the literature related to the information and price predictability of stock markets, this paper finds strong theoretical and empirical support for stock index prediction. Although the introduction of the efficient market hypothesis denied the feasibility of stock index prediction, the emergence of financial market anomalies and the introduction of behavioral finance later confirmed the predictability of stock market. Therefore, it is theoretically and practically feasible to construct a factor database and predict stock index in this paper.

### 2. Stock prediction methods

Stock indices predictive models can be broadly divided into two domains, namely, econometric models and machine learning models.

Classic econometrics models such as ARIMA, VAR and GARCH are widely used in the financial field, and they are mathematically and conceptually well understood. While the econometric framework is characterized by straightforward interpretation of the results and the possibility to perform hypothesis tests, underlying assumptions as linearity, Gaussianity of the errors and stationarity do not hold on stock index data [18–21].

On the other hand, machine learning methods such as Support Vector Machine (SVM) and Gradient Boosted Decision Trees (GBDT) have become popular in stock index prediction due to their accuracy as well as less restrictive underlying assumptions [20, 22, 23]. Within the machine learning domain, deep learning techniques have recently drawn the attention of researchers, principally due to the high accuracy that deep learning models can reach. Deep learning models such as LSTM, Gated Recurrent Unit (GRU) and Deep Neural Network (DNN) have been employed to predict the trend of several stock indices [24–28]. For example, Hoseinzade et al. have proposed a Convolutional Neural Network (CNN) model to predict the stock index trends. Additionally, the authors have validated the CNN based model on financial data drawn from different sources, obtaining in all cases good prediction accuracies [29]. Successively, Eapen et al. have combined first CNN with Bi-LSTM and after CNN with GRU, reaching in both cases very high prediction accuracy on S&P 500 data [30, 31]. Mehtab et al. have proposed four LSTM based models, where each model presents a different architecture. The LSTM models have been employed to predict the NIFTY 50 index values [32]. Nabipour et al. have compared Recurrent Neural Network (RNN) and LSTM to nine standard machine learning methods, showing that the two deep learning models have systematically outperformed the other machine learning methods on continuous data [33]. Livieris et. al have built and trained a Weighted-Constrained DNN (WCDNN) to predict the trend of three stock indices, showing not only the good prediction power of the model but also its numerical efficiency [34].

Most of the deep learning literature on the prediction of stock indices focuses on the improvement of the accuracy of the prediction models, while less attention is devoted to the identification and selection of the driving factors of stock indices dynamics. Within the financial literature, several factors have been elaborated and identified, yet few of them appear to do overlap with already identified factors, and even fewer appear to play a role in the description of asset prices [35]. To tackle the multicollinearity of the existing factors that impact the trend of a stock index, efficient feature selection procedures need to be employed. In detail, feature selection techniques aim to reduce the redundancy of the information within a dataset by selecting only a few of the many available factors. As a result, feature selection techniques reduce factors containing redundant information, while efficiency, accuracy and interpretability of the prediction model. In other words, an efficient feature selection procedure reduces the training time of the model by pulling out of the input dataset redundant factors, while avoiding overfitting of the financial data [28, 36]. Nonetheless the several advantages of feature selection procedures, few studies have applied them in combination with predictive models for stock index trends. Through a careful literature review, we detected three main issues that may hold researchers to apply feature selection procedures: 1) existing techniques require that original data are normalized, a procedure that alters the initial structure of the data with consequent loss of information [37]; 2) Selection techniques return a ranked list of selected factors, while they fail to explain how the features contribute to the stock index dynamics and how have been selected [38–40]; 3) Several studies only apply selection methods on technical indicators, while neglecting micro and macro factors, which may play a pivotal role in a stock index trend [41–43].

To tackle the three issues, we first have constructed a manually curated factors database, which contains not only technical features but also micro and macro indicators. The factor database contains traditional factors such as Price-to-Earnings ratio (P/E) as well as emerging factors as Google Attention Index. Successively, we have selected the relevant factors through TabNet, which can unveil the logic behind the ranking of the features. To do not obscure the natural variability of the information and preserve local and global interpretability, the TabNet procedure has been performed on non-preprocessed data. Finally, we have utilized the resulting important factors to train our predictive LSTM model.

## Stock index trend prediction based on TabNet and LSTM

### 1. Mathematical model on stock index trend prediction

While deep learning models are often regarded as a black-box with good prediction power, in our framework, we identify the factors that characterize the stock index trends, while preserving the high accuracy power of the model. The goal has been reached through the application of TabNet, a feature selection process that selects a minimal number of relevant micro, macro and technical factors, in combination with LSTM, a deep learning model with a strong prediction power. In more detail, the feature selection procedure TabNet permits to select of the most relevant factors from a manually curated database of the economic and financial indicators. The selection of the features allows reducing the complexity of the model training phase while increasing the interpretability of the results. Once the selection procedure is ultimate, the LMST model is trained on the selected factors and successively employed to predict the trends of a stock index. The proposed three-step procedure increases the interpretability of the predictions while preserving the accuracy power of the model.

As a first step, the manual construction of the factors database is performed through a careful data-mining operation across different financial platforms. The mined factors are successively collected on a database and denoted by the n-dimensional set of vectors ${\left\{{\text{x}}_{\text{t}}\right\}}_{\text{t}=1}^{\text{T}}$, where each dimension represent a factor. The factors database consists of a collection micro, macro and technical indicators.

Secondly, the TabNet procedure has been employed on the database ${\left\{{\text{x}}_{\text{t}}\right\}}_{\text{t}=1}^{\text{T}}$ to compute the importance score of each factor. The less relevant factors have been successively filtered out based on a prespecified threshold. The set containing all selected factors is indicated as ${\left\{{\text{x}}_{\text{t}}^{\prime }\right\}}_{\text{t}=1}^{\text{T}}$., where ${\text{x}}_{\text{t}}^{\prime }$ represents the vector of the n′-factors at time t with n′ ≪ n.

Thirdly, the LSTM architecture is designed and trained on ${\left\{{\text{x}}_{\text{t}},{\text{y}}_{\text{t}}\right\}}_{\text{t}=1}^{\text{T}}$, where y_t_ denotes the stock index trend associated at time t. The model is validated on an unseen stock index data.

### 2. TabNet on stock index feature selection

Although feature selection procedures are widely used to predict stock trends, their applications appear to neglect two potential issues that can arise. The first issue regards the preprocessing of the data. Several studies apply the feature selection method after the application of preprocessing strategies on the data, which change the relation across the variables with consequential loss of information. The second issue that arises concerns how the ranking of the importance of the features is performed. Previous studies focused only on the final ranking of features while ignoring how the features have been selected and ranked. As a result, potential confounders may be filtered out from the selection procedure.

To address both issues, Sercan&Pfister have proposed TabNet, a feature selection method published in 2020 and accepted by the Association for the Advance of Artificial Intelligence (AAAI). Compared to other feature selection techniques, TabNet does not require any data preprocessing. Additionally, TabNet does not unveil the importance of each feature at each step of the selection procedure, but it also shows how vital the selected variable is overall. In other words, TabNet helps to give information of each feature’s local and global importance within the selection procedure. Additional computational experiments have shown that TabNet overperforms several existing feature selection procedures on cross-sectional, time-series or panel data [44].

**Fig 1:**
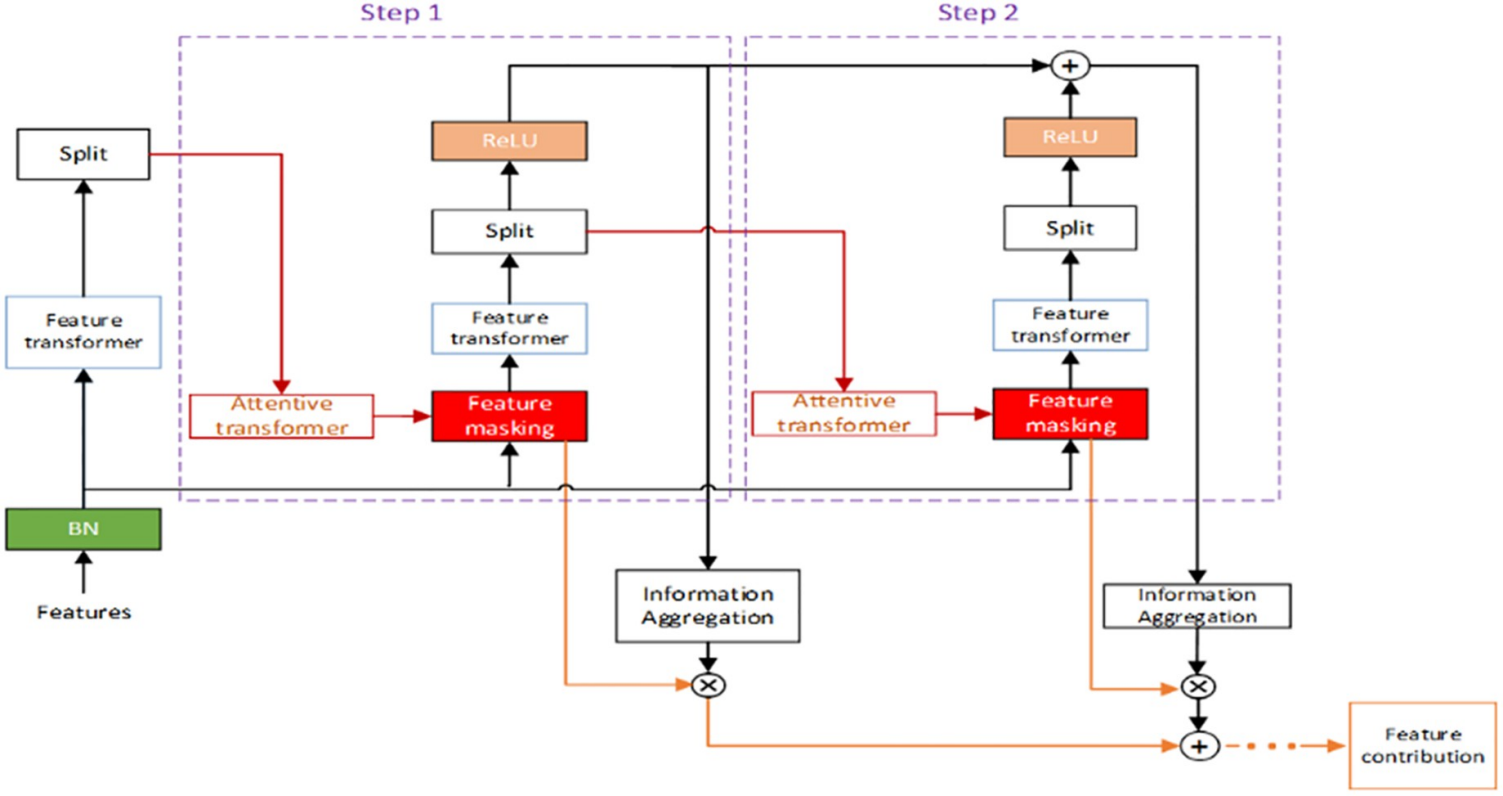
TabNet’s encoding architecture.

In detail, TabNet relies on a sequential multi-step deep neural network architecture, and it returns interpretable informative features that can be employed to train the predictive model. TabNet composes of two types of different architectures, one for encoding and the other for decoding. The TabNet encoder consists of four modules: feature transformer, attentive transformer, feature masking, and split block, and its overall architecture is summarized in Fig 1. As abovementioned, TabNet encoder uses each module results to determine the local importance of each feature and retain the most relevant for the successive modules. The final module elaborates the list of the most relevant features and relative global importance scores. The TabNet decoder comprises a block of feature transformers.

This research applies TabNet feature selection on the manually curated factor database. Only the TabNet encoder architecture is used for the factor selection process.

Specifically, given a factor matrix f ∈ R^B×D^, wherein B is the batch size, D is the number of factors, the steps for selecting factors of the financial market index through TapNet encoding module are as follows:

1. Preliminary feature transformation and data split. The factors are transformed by applying the feature transformer. Successively, the split block provides to divide into batches the transformed data. The split block also extracts the information required by the attentive transformers. Mathematically, this step reads
$$\text{a}\left[0\right]={\text{f}}_{0}\left(\text{f}\right)$$ (1)
  where $\text{a}\left[\text{i}\right]\in {\text{R}}^{\text{B}\times {\text{N}}_{\text{a}}},{\text{f}}_{i}\in {\text{R}}^{\text{B}\times {\text{N}}_{\text{a}}}$ denotes the transformed batch at *i*^*th*^ decision step. At the initial decision step, variables a[0] and f_0_ denotes the required information and transformed batch at the initial decision step.
2. Preliminary attentive transformation. The attentive transformer processes the factor information obtained from the previous step. The following formula can describe the attentive transformation:
$$\text{M}\left[0\right]=\text{sparsemax}\left(\text{P}\left[0\right]\cdot {\text{h}}_{0}\left(\text{a}\left[0\right]\right)\right)$$ (2)
  where sparsemax is a normalization method that encourages sparsity by mapping Euclidean projections to probabilistic simplexes. Variables M[i] ∈ R^B×D^ is a learnable mask for factor selection, and h_i_ is the training function at *i*^*th*^ decision step. The quantity P[i] is a proportional term which indicates the contribution of the corresponding factor. The value P[i] is computed as
$$\text{P}\left[\text{i}\right]=\prod_{\text{j}=1}^{\text{i}}\left(\gamma -\text{M}\left[\text{j}\right]\right)$$ (3)
  where γ denotes a relaxation parameter. When γ = 1, the corresponding factor has contributed only once to the prediction of the stock index trend.
3. Preliminary feature masking. The importance of each factor is computed through the matrix multiplication M[0] · h. The element M_b,j_[0] represents the contribution that factor f_b,j_ has on explaining the stock index trend, and when M_b,j_[0] = 0, the j-th factor in the *b*-th sample does not capture any stock index trends and is consequently filtered out.
4. Feature transformation and data split. A further feature transformer transforms the remaining factors obtained in the previous step. The transformed data are successively divided into d[i], values required for the actual prediction, and a[i], data that undergo a further attentive transformation in the split block. Mathematically, the step can be written as
$$\left[\text{d}\left[\text{i}\right],\text{a}\left[\text{i}\right]\right]={\text{f}}_{\text{i}}\left(\text{M}\left[\text{i}\right]\cdot \text{a}\right)$$ (4)
  Wherein $\text{d}\left[\text{i}\right]\in {\text{R}}^{\text{B}\times {\text{N}}_{\text{d}}}$.
5. Attentive transformation. At this stage, to compute M[i], the a[i] obtained in the previous step are transformed as follows,
$$\text{a}\left[\text{i}-1\right]:\text{M}\left[\text{i}\right]=\text{sparsemax}\left(\text{P}\left[\text{i}-1\right]\cdot {\text{h}}_{\text{i}}\left(\text{a}\left[\text{i}-1\right]\right)\right)$$ (5)
  Wherein where $\sum_{\text{j}=1}^{\text{D}}\text{M}{\left[\text{i}\right]}_{\text{b},\text{j}}=1$.
6. Feature masking. Similarly to the preliminary masking feature step, the multiplication M[i] · i is performed to calculate each factor’s contribution to predicting the stock index trend. Each contribution has been interpreted. The value M_b,j_[i] defines the importance of the factor fbj. When the j-th factor in the b-th sample did not predict the trend, then M_b,j_[i] = 0.
7. Calculation global contribution. After looping steps (4)-(6) for 1000 epochs, the global contribution of the *i*^*th*^ in the *b*-th to the prediction of the stock index trend is denoted as M_agg−b,j_ and calculated as follows.
$${\text{M}}_{\text{agg}-\text{b},\text{j}}=\frac{\sum_{\text{i}=1}^{{\text{N}}_{\text{steps}}}\eta_{\text{b}}\left[\text{i}\right]{\text{M}}_{\text{b},\text{j}}\left[\text{i}\right]}{\sum_{\text{j}=1}^{\text{D}}\sum_{\text{i}=1}^{{\text{N}}_{\text{steps}}}\eta_{\text{b}}\left[\text{i}\right]{\text{M}}_{\text{b},\text{j}}\left[\text{i}\right]}$$ (6)
  Wherein $\eta_{\text{b}}\left[\text{i}\right]=\sum_{\text{c}=1}^{{\text{N}}_{\text{d}}}\text{RELU}\left({\text{d}}_{\text{b},\text{c}}\left[\text{i}\right]\right)$, which represents the factor contribution at i-th decision step in the b-th sample. When d_b,c_[i] < 0, the contribution of all factors at i-th decision step is zero. In addition, $\sum_{\text{j}=1}^{\text{D}}{\text{M}}_{\text{agg}-\text{b},\text{j}}=1$.

### 3. LSTM on stock index trend prediction

In deep learning, the Recurrent Neural Network (RNN) is a neural network model known for its reliable prediction on classification problems when the input data are time series. In particular, the chain structure of the RNN shows good performance also in the case the time series are sparse. On the other hand, RNN can only retain short sequences due to well-known cons such as gradient disappearance and gradient explosion [45].

**Fig 2:**
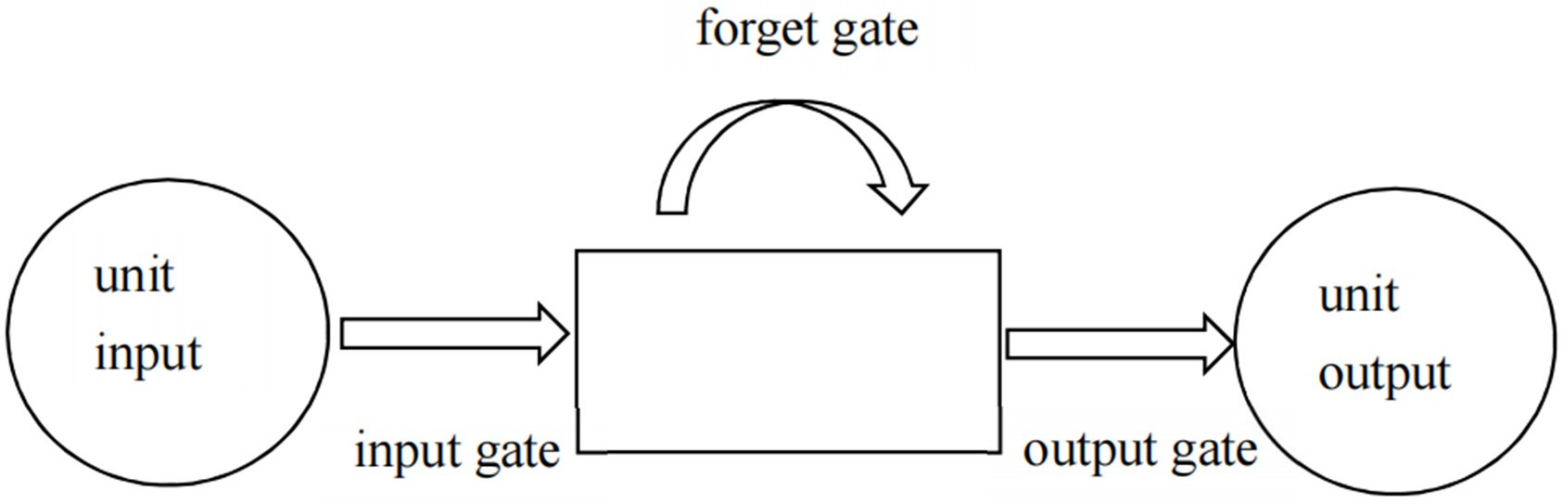
Storage unit architecture.

In light of the pros and cons of the RNN, here, the RNN has been employed to predict future trends of the stock index from the historical factor data. Here, we utilized the Long Short-Term Long Memory (LSTM) model, which has the advantage to store more extended sequences of information compare to the classic RNN. In particular, an introduction of a storage unit gives the LSTM higher memory capacity. The storage unit consists of four parts: 1) an input gate; 2) a forget gate; 3) an output gate; 4) a self-circulating neuron. The overall structure of the storage unit is presented in Fig 2.

**Fig 3:**
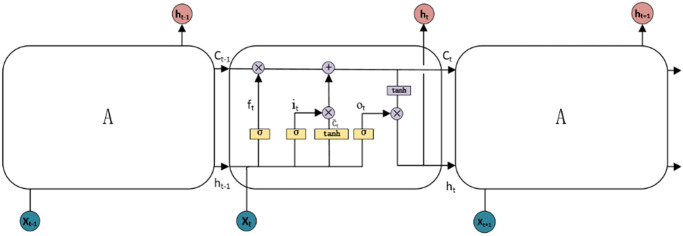
LSTM’s expanded network form of the storage unit.

In the storage unit, gates control the interaction amongst adjacent units. The input gate selects all the information that needs to be processed or retained. The selected data undergo an S-shaped and a tanh layer. The model decides if the inputs can change their state within the storage unit from the input gate. Successively, the forget state can choose to consider or not some information. The forget gate returns a continuous value in a unit range, where the "0" means a previous state is "completely ignored", while "1" that the previous state is "completely reserved". The output gate computes the final results drawn by considering the outputs of each storage unit. In other words, the output results are determined by the unit state, filtered data and newly added information. The storage unit in Fig 3 represents the architecture of the augmented LSTM model and how each gate contributes to the prediction generated by the model [46].

Mathematically, the LSTM architecture can be described as below.


$${\text{i}}_{\text{t}}=\sigma \left({\text{W}}_{\text{i}}{\text{x}}_{\text{t}}+{\text{U}}_{\text{i}}{\text{h}}_{\text{t}-1}+{\text{b}}_{\text{i}}\right)$$ (7)

$${\tilde{\text{C}}}_{\text{t}}=\text{tanh}\left({\text{W}}_{\text{c}}{\text{x}}_{\text{t}}+{\text{U}}_{\text{c}}{\text{h}}_{\text{t}-1}+{\text{b}}_{\text{c}}\right)$$ (8)

$${\text{f}}_{\text{t}}=\sigma \left({\text{W}}_{\text{f}}{\text{x}}_{\text{t}}+{\text{U}}_{\text{f}}{\text{h}}_{\text{t}-1}+{\text{b}}_{\text{f}}\right)$$ (9)

$${\text{C}}_{\text{t}}={\text{i}}_{\text{t}}*{\tilde{\text{C}}}_{\text{t}}+{\text{f}}_{\text{t}}{*\text{C}}_{\text{t}-1}$$ (10)

$${\text{o}}_{\text{t}}=\sigma \left({\text{W}}_{\text{o}}{\text{x}}_{\text{t}}+{\text{V}}_{\text{o}}{\text{h}}_{\text{t}-1}+{\text{b}}_{\text{o}}\right)$$ (11)

$${\text{h}}_{\text{t}}={\text{o}}_{\text{t}}*\text{tanh}\left({\text{C}}_{\text{t}}\right)$$ (12)

Here, W_i_, W_c_, W_f_, W_o_, U_i_, U_c_, U_f_, U_o_, V_o_ are the weight matrices, while b_i_, b_c_, b_f_, b_o_ are the deviation vectors. The variables x_t_ and h_t_ are respectively input and output vectors of the storage unit at time t. Furthermore, variables i_t_, f_t_, o_t_ are variables of the input, forget, and output gate, while ${\tilde{\text{C}}}_{\text{t}}$ and C_t_ represent the candidate state and the state of the storage unit time t.

The TabNet procedure combined with the LSTM model are employed to predict the stock market trends by using the time series of different factors. The resulting factor selected from an initial manually curated database through TabNet can unveil the driving dynamics of the stock index trend and increase the prediction power of the LSTM model.

## Data

### 1. Data set and data preprocessing

This study evaluates the performance of the TabLSTM through its application on four different stock indices, namely Dow Jones Composite Index, S&P 500, China Shanghai Composite Index (00001), and China Shenzhen Composite Index (399106). Each index has been considered from 01/02/2008 to 11/19/2020. The Dow Jones Composite Index and S&P 500 are two stock indices of the US financial market, a benchmark for developed countries. On the other hand, the China Shanghai Composite Index and the China Shenzhen Composite Index are two representative stock indices of the Chinese financial market, a potential benchmark for developing countries. Specifically, the China Shanghai Composite Index reflects the price movement of stocks listed on the Shanghai Stock Exchange, while the China Shenzhen Composite Index reflects the combined price movement of all stocks listed on the Shenzhen Stock Exchange. The data have been collected through different certified resources, such as Wind Financial Data Database, containing 3133 daily transaction information.

The data with small variability have been filtered out based on a lower and upper threshold. The lower threshold is set equal to -0.50%, while the upper bound is 0.55%. In other words, only financial out of the two thresholds are considered. Additional two filters have been applied. One filter erases data associated with null trading volume, and the second filters out the factors with more than 50% missing values. After the application of the three filters, the clean data contains 2524 daily observations. The clean data are successively split into three sets, namely training set (80% of the observations), validation set (10% of the observations) and test set (10% of the observations). The training set is used for feature selection and prediction model training, the validation set is used for hyperparameter tuning, and the test set is used to evaluate the predictive power of the model.

The manually curated factor database utilized as input information for TabNet contains thirty-five macroeconomic factors [47–49], seven microeconomic factors [50–52] and seventy-two technical factors [53–56]. The identification of the factor is a result of a careful review of the financial and economic literature (see S1–S3 Tables). The prediction of the stock index trend has been rewritten as a classification problem. The trend variable, also known as binary movement, is based on the thirty-day difference between two close prices. If the stock difference records a loss, then "0" is attributed to the binary movement, while "1" denotes if the difference signs a profit. The binary movement is denoted as y = I(P_t+30_ > P_t_), where I(·) is the indicator function.

### 2. Evaluation metrics

The prediction performance of the TabLSTM model has been evaluated using three different indicators, namely Area Under Roc Curve (AUC), Balanced Accuracy, and Error Rate. The first two metrics measure the prediction accuracy of a model, while the last metric measures the prediction error of a model. The mathematical description of each metric is given in Table 1. The prediction performance of the proposed model is compared to the performance of six models, namely, XGBoost [57], LightGBM [58], GBDT [59], CatBoost [60], Logitstic Regression [61], and K-NearestNeighbor (KNN) [62]. In particular, XGBoost, LightGBM, GBDT, and CatBoost are state of art classification models with feature selection function, while LR and KNN are traditional classification models without feature selection function.

**Table 1:** Evaluation metrics and their calculations.

Metrics	Calculations
Auc	$\text{AUC}=\frac{\sum_{ins_{i}\in positiveclass}\;rank_{ins_{i}}-\frac{M\times \left(M+1\right)}{2}}{M\times N}$
Balanced Accuracy	$\text{Balanced}\_\text{Accuracy}\left(\text{y},{\text{y}}^{\prime }\right)=\frac{1}{\text{k}}\sum_{\text{i}=1}^{\text{k}}\sum_{\text{j}=1}^{{\text{m}}_{\text{i}}}1\left({\text{y}}_{\text{ij}}^{\prime }={\text{y}}_{\text{ij}}\right)$
Error Rate	$\text{Error}\_\text{Rate}\left(\text{y},{\text{y}}^{\prime }\right)=1-\frac{1}{\text{n}}\sum_{\text{i}=1}^{\text{n}}1\left({\text{y}}_{\text{i}}^{\prime }={\text{y}}_{\text{i}}\right)$

In Table 1, *ins*_*i*_ is the i-th sample, $rank_{ins_{i}}$ is the rank position of the i-th sample in a ascending ordering. M and N are the number of positive samples and that of negative samples, respectively. Variables y, y’ are respectively the actual and predicted binary movements of the stock index. The index *k* represents the number of categories, in this study *k* = 2, while m_i_ and n are the respective numbers of samples in each category.

## Results and discussion

The prediction of the stock index trends based on a factor database, TabNet feature selection and LSTM method is applied on the four indices, Dow Jones Composite Index, S&P 500, China Shenzhen Composite Index, and China Shanghai Composite Index. For the TabNet feature selection, the dimensions of *a*[i] and *d*[i], which are denoted as N_a_ and N_d_, are set to be 8. The decision steps N_step_ = 3, sparsity coefficient λ_sparse_ = 0.001, a relaxation parameter in preliminary attentive transformation step γ = 1.3, a small number for numerical stability in sparsity regularization ε = 1 × 10^−15^, batch size *B* = 128, learning rate = 0.02 and iteration = 1000. For the LSTM, the number of neurons in the hidden layer and output layer are arbitrarily chosen to be 128 and 1 respectively, dropout = 0.2 and iteration = 100. The optimization of the parameter is performed by using Adam optimizer. The code is implemented in Python 3.

### 1. Stock index feature selection

**Fig 4:**
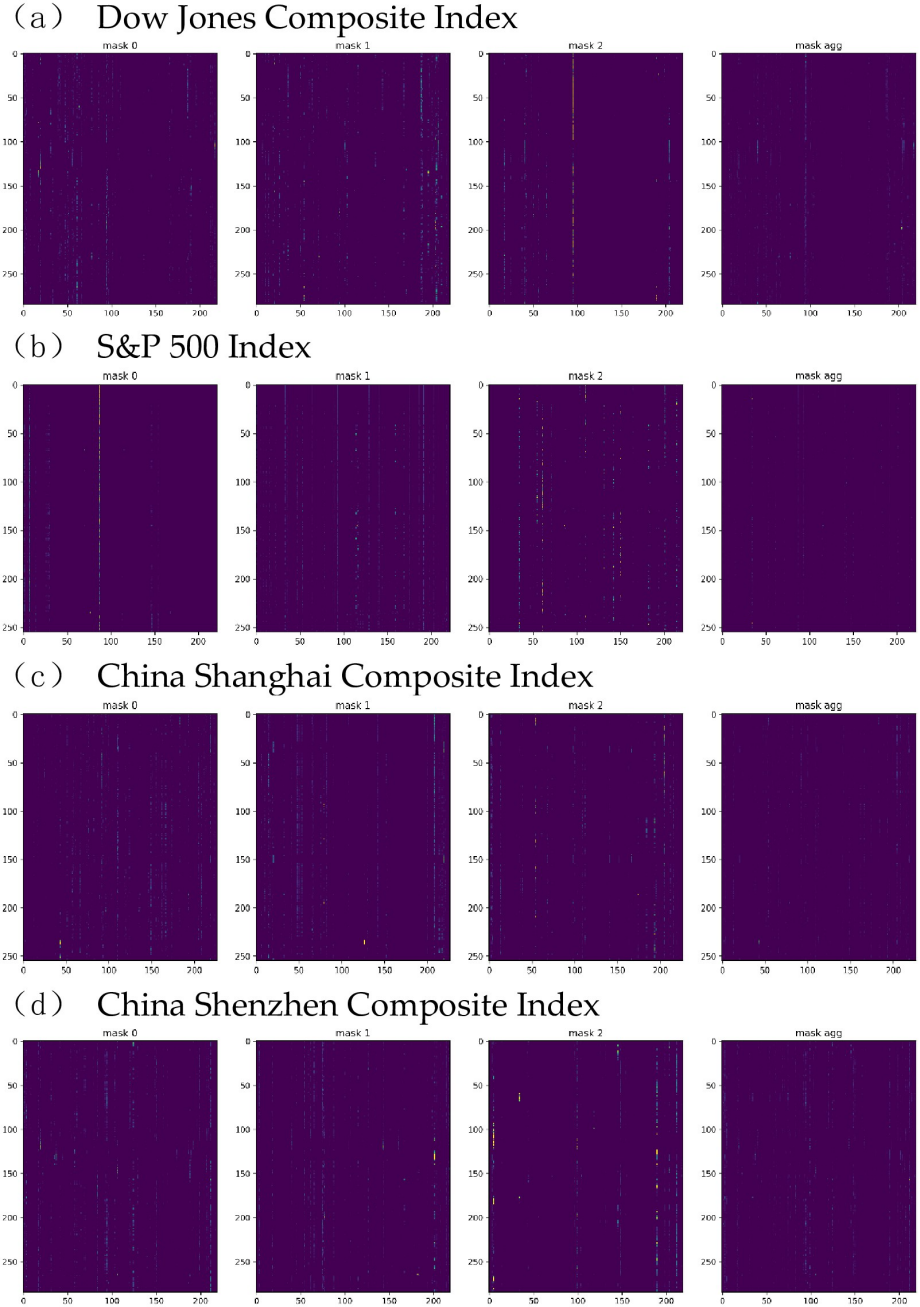
Factor importance ranking. (a) Dow Jones Composite Index. (b) S&P 500 Index. (c) China Shanghai Composite Index. (d) China Shenzhen Composite Index.

The TabNet encoder’s application leads to identifying several factors. Fig 4 shows feature importance masks *mask*[*i*] that indicates feature selection at (*i* + 1)^*th*^ decision step, and its aggregate feature importance M_agg_ that indicates the outcomes of global feature selection. The x-axis and y-axis in each subfigure of Fig 4 represent the factor number, and the decision step, respectively and the bright light highlights the factors with high importance. For example, in the first decision step which denoted as mask[0], the factors numbered between 0 and 100 show more feature importance than the factors numbered after 100. In the second decision step which denoted as mask[1], factors numbered around 200 are more importance than the factors numbered between 0 and 100. In the third decision step which denoted as mask[2], factors numbered around 100 show more significant feature importance than other factors. In the aggregate feature selection step which denoted as mask agg, the aggregate masks for factors numbered between 100 and 200 are almost all zero, indicating that these factors are irrelevant to predict Dow Jones Composite Index. Moreover, factors numbered between 0 and 100, and factors numbered around 200 play a role in predicting Dow Jones Composite Index. The outcomes of the aggregate mask are consistent with the previous decision steps. Furthermore, these results show that Tabnet can merely focus on the relevant factors and produce accurate feature selection results.

Moreover, Table 2 presents the top ten factors with the highest importance score for each index. As can be seen in Table 2, the most influential factors in each index overlap considerably. Ignoring the specific factor ranking, the nine factors, namely P/E, P/CF, P/D, B/M, D/E, LEVERAGE, MOMENTUM 1 WEEK, MOMENTUM 2 WEEK, and MOMENTUM 3 WEEK are located in the top ten impact factors of all indices. In addition, Table 2 shows that the factors that have the greatest impact on all indices fall under the category of micro and technical factors.

**Table 2:** Top ten factors for composite indices.

Rank	Dow Jones Composite Index	S&P 500 Index	China Shanghai Composite Index	China Shenzhen Composite Index
1	P/E	P/E	P/E	P/E
2	P/CF	P/CF	P/CF	P/CF
3	P/D	P/S	P/S	P/S
4	B/M	P/D	P/D	P/D
5	D/E	B/M	B/M	B/M
6	LEVERAGE	D/E	D/E	D/E
7	MOMENTUM 1 WEEK	LEVERAGE	LEVERAGE	LEVERAGE
8	MOMENTUM 2 WEEK	MOMENTUM 1 WEEK	MOMENTUM 1 WEEK	MOMENTUM 1 WEEK
9	MOMENTUM 3 WEEK	MOMENTUM 2 WEEK	MOMENTUM 2 WEEK	MOMENTUM 2 WEEK
10	MOMENTUM 1 MONTH	MOMENTUM 3 WEEK	MOMENTUM 3 WEEK	MOMENTUM 3 WEEK

### 2. Stock index trend prediction

After obtaining the feature importance of each stock index, the LSTM model is trained on the highest-ranked factors, and the resulting model is used to predict the stock market trends. Since the ten highest-ranked factors in each stock index can already explain nearly 60% of stock index movement, this paper chooses these ten highest-ranked factors in each stock index for stock index trend prediction. The prediction performance and convergence time of all the models, including TabLSTM, are shown in Tables 3 and 4, respectively.

**Table 3:** Prediction results of different methods.

(a) Dow Jones Composite Index				
**Database**	**Methods**	**Auc**	**Balanced Accuracy**	**Error Rate**
Factor Database	TabLSTM	**0.9279**	**0.8635**	**0.1225**
	XGBoost	0.8733***	0.8078	0.2119
	LightGBM	0.8616***	0.7922	0.2221
	GBDT	0.8503***	0.7529	0.2798
	CatBoost	0.8805***	0.8275	0.1920
	Logistic Regression	0.5576***	0.7176	0.4603
	KNN	0.6899***	0.6863	0.3503
Technical Indicators	TabLSTM	0.5811***	0.5626	0.3725
(b) S&P 500 Index				
**Database**	**Methods**	**Auc**	**Balanced Accuracy**	**Error Rate**
Factor Database	TabLSTM	0.9008	**0.8778**	**0.1147**
	XGBoost	**0.9230** **	0.8421	0.1581
	LightGBM	0.9227**	0.8491	0.1512
	GBDT	0.8890***	0.8386	0.1624
	CatBoost	0.9175***	0.8386	0.1624
	Logistic Regression	0.6339***	0.6246	0.3793
	KNN	0.8034***	0.7614	0.2394
Technical Indicators	TabLSTM	0.7798***	0.6877	0.2627
(c) China Shanghai Composite Index				
**Database**	**Methods**	**Auc**	**Balanced Accuracy**	**Error Rate**
Factor Database	TabLSTM	**0.9343**	**0.8778**	**0.1228**
	XGBoost	0.9137***	0.8386	0.1622
	LightGBM	0.9061***	0.8281	0.1726
	GBDT	0.8791***	0.8035	0.1978
	CatBoost	0.9162***	0.8421	0.1585
	Logistic Regression	0.7348 ***	0.6912	0.3155
	KNN	0.7807***	0.7298	0.2700
Technical Indicators	TabLSTM	0.6172***	0.5833	0.4281
(d) China Shenzhen Composite Index				
**Database**	**Methods**	**Auc**	**Balanced Accuracy**	**Error Rate**
Factor Database	TabLSTM	**0.9132**	0.8428	**0.1649**
	XGBoost	0.8977***	0.8275	0.2088
	LightGBM	0.8979***	0.8196	0.2118
	GBDT	0.8672***	0.7961	0.2441
	CatBoost	0.9129**	**0.8510**	0.1853
	Logistic Regression	0.7340***	0.7647	0.3391
	KNN	0.8021***	0.7686	0.2735
Technical Indicators	TabLSTM	0.6092***	0.5904	0.4246

**and*** indicate statistical significance at the 5% and 10% level based on the z-test, respectively.

**Table 4:** Convergence time(s) of different methods.

Methods	Dow Jones Composite Index	S&P 500 Index	China Shanghai Composite Index	China Shenshen Composite Index
TabLSTM	85.3028	158.2536	149.7281	176.0523
XGBoost	193.6833	169.2504	248.9865	297.3121
LightGBM	134.0416	138.0898	144.8090	145.9992
GBDT	190.8409	124.9596	143.7918	184.1390
CatBoost	**163.2196**	**108.2852**	**142.5821**	**180.4363**
Logistic Regression	19.1367	20.0425	20.9373	22.7392
KNN	**0.2991**	**0.3036**	**0.7449**	**0.5898**
TabLSTM	85.3028	158.2536	149.7281	176.0523

Table 3 summarizes the predictive performance of all methods, including XGBoost, LightGBM, GBDT, CatBoost, Logistic Regression, and KNN. The best results for these methods are highlighted in bold face in Table 3. The total count of the bold-faced results is 10 for TabLSTM and 2 for other methods, which indicates the relative predictive power of TabLSTM.

Generally, a forecasting accuracy of 56% is satisfactory in stock trend prediction (Haq et al, 2021). Table 3 shows that the TabLSTM model combined with a factor database achieves satisfying results in Dow Jones Composite Index, S&P 500 Index, China Shanghai Composite Index, and China Shenshen Composite Index. Specifically, in the Dow Jones Composite Index, the TabLSTM model has an auc of 92.79%, balanced accuracy of 86.35%, and error rate of 12.25%. In the S&P 500 Index, the TabLSTM model has an auc of 90.08%, balanced accuracy of 87.78%, and error rate of 11.47%. In the China Shanghai Composite Index, the TabLSTM model can achieve an auc of 93.43%, balanced accuracy of 87.78%, and error rate of 12.28%. In the China Shenzhen Composite Index, the TabLSTM model can achieve an auc of 91.32%, balanced accuracy of 84.28%, and error rate of 16.49%. These results all prove the good prediction performance of the proposed method.

In addition, TabLSTM model perform much better than other trend prediction models, especially the traditional models. Take Dow Jones Composite Index as an example, TabLSTM model outperforms four state of art classification models (XGBoost, LightGBM, GBDT, CatBoost) by 6.25%, 7.69%, 9.13%, 5.38% in auc metric, 6.90%, 9.00%, 14.69%, 4.35% in balanced accuracy metric, 42.19%, 44.84%, 56.22%, 36.20% in error rate metric. Moreover, TabLSTM model significantly outperforms two traditional classification models (Logistic Regression and KNN) by 66.41% and 34.50% in auc metric, 20.33% and 25.82% in balanced accuracy metric, 73.39% and 65.03% in error rate metric, suggesting superior performance of the proposed method further.

Moreover, Table 3 also shows the prediction results of the TabLSTM method based on the proposed factor database and technical indicators, respectively. Obviously, we could obtain better performance results based on a comprehensive factor database rather than a pool of technical indicators. Take Dow Jones Composite Index as an example, TabLSTM model with factor database outperforms that with technical indicators by 59.68%, 53.48%, 67.11% in auc, balanced accuracy, error rate, respectively, suggesting the necessity to construct a comprehensive factor database.

Table 4 summarizes the convergence time of all methods in seconds. The shortest convergence time of state of art classification models (XGBoost, LightGBM, GBDT, CatBoost) and traditional classification models (Logistic Regression, KNN) are highlighted in bold face, respectively. Generally, TabLSTM and four state of art classification models with better prediction performance require more time to converge than the two traditional classification models. Moreover, TabLSTM with the best prediction performance doesn’t always show the shortest convergence time, which indicates that TabLSTM require further optimization for better applications.

## Conclusion

In this paper, we propose a novel hybrid model, TabNet, which combines LSTM with a feature selection technique, TabNet, to predict the stock index trend. The hybrid model includes three components: constructing factor library, selecting relevant features and predicting stock index trend. In the first phase, we summarize the potential factors from the existing literature and construct a factor library containing micro, macro and technical factors. In the second phase, TabNet encoder is trained to identify the driving factors of the stock index trend, in which we could also learn the local and global importance of each feature. In the last phase, we use the highest-ranked factors as the inputs of a LSTM model to predict the 30-day-ahead stock index movements.

In the empirical study, we conduct an experiment to test the prediction performance of the TabLSTM model. Take four stock indices as research objects, namely, Dow Jones Composite Index, S&P 500, Shanghai Composite Index, and Shenzhen Composite Index, the performance of TabLSTM is compared to the other six models, including XGBoost, LightGBM, GBDT, CatBoost, Logitstic Regression, and K-NearestNeighbor (KNN). The results show that our hybrid prediction model with a higher auc value, a higher balanced accuracy value and a lower error rate is superior to the traditional classification models and other state of art classification models proposed by the previous literature. Moreover, our hybrid prediction model with a comprehensive factor library outperforms the prediction model with only technical factors in terms of all evaluation metrics. We conclude that the construction of a comprehensive factor library based on the TabLSTM feature selection technique and LSTM model can build a prediction model that brings better prediction performance for stock index trend.

In summary, the contributions of the proposed model, TabLSTM, can be found in three aspects. The TabLSTM model that combines LSTM with a feature selection model, TabNet, provides a comprehensive understanding for predicting stock index trend from feature selection level and the trend prediction level. The factor library constructing stage provides potential factors from macro level, micro level and technical level to support further analysis of stock index trend prediction. In addition, the TabNet encoder helps to complement the TabLSTM model by analyzing the local feature importance and global feature importance of each factor in the original data. Furthermore, it is our hope that the process to build the TabLSTM model provides an example for predicting stock index trend in a more comprehensive manner.

However, there are still four possible extensions in this study. First of all, the research can include data from more stock markets for experiment and robustness test. Secondly, the model can be extended to a broader range of financial products, such as commodities, bonds, digital currencies etc. Thirdly, the manually curated financial factor library can be to extended to include more factors that may influence the trend of stock index, thereby improving the prediction performance of the proposed method. Lastly, TabNet could be optimized shorten its convergence time.

## Supporting information

S1 Table Macro factor description. (DOCX)

S2 Table Micro factor description. (DOCX)

S3 Table Technical factor description. (DOCX)
