# Supplementary material for: Stock index trend prediction based on TabNet feature selection and long short-term memory
Source: PLoS One. 2022 Dec 13;17(12):e0269195. doi: 10.1371/journal.pone.0269195 (PMC9746941; doi:10.1371/journal.pone.0269195)
Supplement: S1 Table — (DOCX) [file pone.0269195.s001.docx]

S1 Table. Macro factor description

| **Number** | **Classification** | **Factor** | **Description** |
| --- | --- | --- | --- |
| 1 | Cross-asset factor | CRUDE | Crude price |
| 2 | Cross-asset factor | GOLD | Gold price |
| 3 | Cross-asset factor | GP | Gold-platinum ratio |
| 4 | Cross-asset factor | BOND | 5-year bond index yield |
| 5 | Cross-asset factor | INVBOND SPREAD | The daily price difference between Baa-rated corporate bonds and 10-year government bonds |
| 6 | Cross-asset factor | COPBOND SPREAD | The daily price difference between AAA corporate bonds and BAA corporate bonds |
| 7 | Cross-asset factor | TREASURY RATE | The second market interest rate of the 3-month Treasury bill |
| 8 | Cross-asset factor | TREASURY SPREAD | Daily spread between the 3-month Treasury bill and the 10-year national bond |
| 9 | Cross-asset factor | LONTREASURY YIELD | Long-term state bond yield |
| 10 | Cross-asset factor | CHF/CNY (or USD) | The exchange rate between the Swiss franc and the currency of an index-located country |
| 11 | Cross-asset factor | EUR/CNY (or USD) | The exchange rate between the Eurodollar and the currency of an index-located country |
| 12 | Cross-asset factor | GBP/CNY (or USD) | The exchange rate between the British Pound and the currency of an index-located country |
| 13 | Cross-asset factor | JPY/CNY (or USD) | The exchange rate between the Japanese Yen and the currency of an index-located country |
| 14 | Cross-asset factor | USD/CNY | The exchange rate between the U.S. dollar and RMB |
| 15 | Cross-asset factor | WEIGHTED EXT | Trade-weighted exchange rate |
| 16 | National economic factor | GOOGLE ATTEN | Daily search volume of the country term in the Google search engine |
| 17 | National economic factor | GDP | — |
| 18 | National economic factor | INFLATION | — |
| 19 | National economic factor | CPI | — |
| 20 | National economic factor | IP | Industrial Production Index |
| 21 | National economic factor | PMI | Purchasing Manager Index |
| 22 | National economic factor | LEI | Council Leading Economic Index |
| 23 | National economic factor | OUTPUT GAP | — |
| 24 | National economic factor | MONEY SUPPLY | The monthly change rate of money supply (M1) |
| 25 | National economic factor | CAY | The ratio of non-durable goods consumption to total wealth (including labour and non-labour) |
| 26 | National economic factor | KI | Killian Index |
| 27 | International economic factors | ADS | US Aruoba–Diebold–Scotti Economic Condition Index |
| 28 | International economic factors | CFNAI | Chicago Fed Monthly National Activity Index |
| 29 | International political factors | USELEC 6M | If the month is within 6 months before the U.S. federal election, the value is 1, otherwise, it is 0 |
| 30 | International political factors | USELEC 3M | If the month is within 3 months before the U.S. federal election, the value is 1, otherwise, it is 0 |
| 31 | International political factors | USELEC 1M | If the month is within 1 month before the U.S. federal election, the value is 1, otherwise, it is 0 |
| 32 | International political factors | CHIELEC 6M | If the month is within 6 months before the change of Chinese leadership, the value is 1, otherwise, it is 0 |
| 33 | International political factors | CHIELEC 3M | If the month is within 3 months before the change of Chinese leadership, the value is 1, otherwise, it is 0 |
| 34 | International political factors | CHIELEC 1M | If the month is within 1 month before the change of Chinese leadership, the value is 1, otherwise, it is 0 |
| 35 | National social factor | AQI | — |
