# Supplementary material for: Stock index trend prediction based on TabNet feature selection and long short-term memory
Source: PLoS One. 2022 Dec 13;17(12):e0269195. doi: 10.1371/journal.pone.0269195 (PMC9746941; doi:10.1371/journal.pone.0269195)
Supplement: S2 Table — (DOCX) [file pone.0269195.s002.docx]

S2 Table. Micro factor description

| **Number** | **Factor** | **Description** |
| --- | --- | --- |
| 36 | P/E | The ratio of market price to earnings per share |
| 37 | P/CF | The ratio of market price to cash flow per share |
| 38 | P/S | The ratio of market price to sales per share |
| 39 | P/D | The ratio of market price to dividend per share |
| 40 | B/M | The logarithm ratio of shareholders' equity to the company's market value |
| 41 | D/E | The ratio of total liabilities to shareholders' equity |
| 42 | LEVERAGE | The ratio of total liabilities to total asset |
