# Supplementary material for: Stock index trend prediction based on TabNet feature selection and long short-term memory
Source: PLoS One. 2022 Dec 13;17(12):e0269195. doi: 10.1371/journal.pone.0269195 (PMC9746941; doi:10.1371/journal.pone.0269195)
Supplement: S3 Table — (DOCX) [file pone.0269195.s003.docx]

S3 Table. Technical factor description

| **Number** | **Classification** | **Factor** | **Description** |
| --- | --- | --- | --- |
| 43 | Momentum and reversal factor | MOMENTUM 1WEEK | Percentage change of stock yield in 1 week |
| 44 | Momentum and reversal factor | MOMENTUM 2WEEK | Percentage change of stock yield in 2 weeks |
| 45 | Momentum and reversal factor | MOMENTUM 3WEEK | Percentage change of stock yield in 3 weeks |
| 46 | Momentum and reversal factor | MOMENTURM 1MONTH | Percentage change of stock yield in 1 month |
| 47 | Momentum and reversal factor | MOMENTURM 2MONTH | Percentage change of stock yield in 2 months |
| 48 | Momentum and reversal factor | MOMENTURM 3MONTH | Percentage change of stock yield in 3 months |
| 49 | Momentum and reversal factor | MOMENTURM 4MONTH | Percentage change of stock yield in 4 months |
| 50 | Momentum and reversal factor | MOMENTURM 6MONTH | Percentage change of stock yield in 6 months |
| 51 | Momentum and reversal factor | MOMENTURM 11MONTH | Percentage change of stock yield in 11 months |
| 52 | Momentum and reversal factor | MOMENTURM 1YEAR | Percentage change of stock yield in 1 year |
| 53 | Momentum and reversal factor | MOMENTURM 3YEAR | Percentage change of stock yield in 2 years |
| 54 | Momentum and reversal factor | MOMENTURM 4YEAR | Percentage change of stock yield in 4 years |
| 55 | Momentum and reversal factor | MOMENTURM 5YEAR | Percentage change of stock yield in 5 years |
| 56 | Momentum and reversal factor | industry momentumy | Percentage change of industry sub-index yield in 1 year |
| 57 | Momentum and reversal factor | industry momentumm1 | Percentage change of industry sub-index yield in 6 months |
| 58 | Momentum and reversal factor | industry momentumm2 | Percentage change of industry sub-index yield in 1 month |
| 59 | Momentum and reversal factor | industry momentumw | Percentage change of industry sub-index yield in 1 week |
| 60 | Momentum and reversal factor | returny1 | Monthly return 3 years ago |
| 61 | Momentum and reversal factor | returny2 | Monthly return 2 years ago |
| 62 | Momentum and reversal factor | returny3 | Monthly return 1 year ago |
| 63 | Liquidity factor | TURN | The ratio of trading volume to the number of outstanding shares |
| 64 | Liquidity factor | DTURNy | The ratio of daily trading volume to the average number of shares outstanding in the past 365 days |
| 65 | Liquidity factor | DTURNm | The ratio of daily trading volume to the average value of shares outstanding in the past 30 days |
| 66 | Liquidity factor | DTURNw | The ratio of daily trading volume to the average value of shares outstanding in the past 7 days |
| 67 | Liquidity factor | PRCVOLy | The logarithmic average of daily turnover within 365 days |
| 68 | Liquidity factor | PRCVOLm | The logarithmic average of daily turnover within 30 days |
| 69 | Liquidity factor | PRCVOLw | The logarithmic average of daily turnover within 7 days |
| 70 | Liquidity factor | VOLSCALED | The logarithmic average ratio of daily turnover to the company's market value within 7 days |
| 71 | Liquidity factor | MARKETCAP | The Logarithm of turnover |
| 72 | Liquidity factor | PRC | The logarithm of stock price |
| 73 | Liquidity factor | SPREAD | The difference between the bid and ask |
| 74 | Liquidity factor | DVOL | The natural logarithm of the U.S. dollar volume on the penultimate trading day |
| 75 | Liquidity factor | VOLy | The logarithmic average of daily volume within 365 days |
| 76 | Liquidity factor | VOLm | The logarithmic average of daily volume within 30 days |
| 77 | Liquidity factor | VOLw | The logarithmic average of daily volume within 7 days |
| 78 | Liquidity factor | STDVOLy1 | The natural logarithm of daily volume’s standard deviation within 3 years |
| 79 | Liquidity factor | STDVOLy2 | The natural logarithm of daily volume’s standard deviation within 1 year |
| 80 | Liquidity factor | STDVOLm | The natural logarithm of daily volume’s standard deviation within 30 days |
| 81 | Liquidity factor | STDVOLw | The natural logarithm of daily volume’s standard deviation within 7 days |
| 82 | Liquidity factor | ILLIQ | The average ratio of each stock's return to trading volume |
| 83 | Liquidity factor | DTURN | The natural logarithm of turnover rate, wherein the turnover rate is calculated as the ratio of trading volume to the number of outstanding shares |
| 84 | Liquidity factor | STDTURNy1 | The natural logarithm of turnover rate’s standard deviation in 3 years |
| 85 | Liquidity factor | STDTURNy2 | The natural logarithm of turnover rate’s standard deviation in 365 days |
| 86 | Liquidity factor | STDTURNm | The natural logarithm of turnover rate’s standard deviation in 30 days |
| 87 | Liquidity factor | STDTURNw | The natural logarithm of turnover rate’s standard deviation in 7 days |
| 88 | Liquidity factor | COVRETURNy | Covariance of price change rate within 365 days |
| 89 | Liquidity factor | COVRETURNm | Covariance of price change rate within 30 days |
| 90 | Liquidity factor | COVRETURNw | Covariance of price change rate within 7 days |
| 91 | Volatility factor | RETURN VOLy | The standard deviation of daily yield within 365 days |
| 92 | Volatility factor | RETURN VOLm | The standard deviation of daily yield within 30 days |
| 93 | Volatility factor | RETURN VOLw | The standard deviation of daily yield within 7 days |
| 94 | Volatility factor | VIX | The weighted average of S&P 500 call options and put option's implied volatility |
| 95 | Volatility factor | KURTOSISy | The kurtosis of daily yield within 365 days |
| 96 | Volatility factor | KURTOSISm | The kurtosis of daily yield within 30 days |
| 97 | Volatility factor | KURTOSISw | The kurtosis of daily yield within 7 days |
| 98 | Volatility factor | SKEWNESSy | The skewness of daily yield within 365 days |
| 99 | Volatility factor | SKEWNESSm | The skewness of daily yield within 30 days |
| 100 | Volatility factor | SKEWNESSw | The skewness of daily yield within 7 days |
| 101 | Volatility factor | MAXRETm | The highest value of daily yield within 30 days |
| 102 | Volatility factor | MAXRETw | The highest value of daily yield within 7 days |
| 103 | Volatility factor | MINRETm | The lowest value of daily yield within 30 days |
| 104 | Volatility factor | MINRETw | The lowest value of daily yield within 7 days |
| 105 | Volatility factor | DAMIHUDm | The average ratio of yield to volume in 30 days |
| 106 | Volatility factor | DAMIHUDw | The average ratio of yield to volume in 7 days |
| 107 | Volatility factor | VOLy | $\frac{\mathrm{high}_{365}-\mathrm{low}_{365}}{\left( \mathrm{high}_{365}+\mathrm{low}_{365} \right)/2}$ |
| 108 | Volatility factor | VOLm | $\frac{\mathrm{high}_{30}-\mathrm{low}_{30}}{\left( \mathrm{high}_{30}+\mathrm{low}_{30} \right)/2}$ |
| 109 | Volatility factor | VOLw | $\frac{\mathrm{high}_{7}-\mathrm{low}_{7}}{\left( \mathrm{high}_{7}+\mathrm{low}_{7} \right)/2}$ |
| 110 | Technical transaction rules | MOM | $S_{t,MOM}=\left\{ \begin{aligned} 1,P_{t}\geq P_{t-k} \\ 0,else \end{aligned} \right.$ |
| 111 | Technical transaction rules | FR | $S_{t,FR}^{\mathrm{buy}}=\left\{ \begin{aligned} 1,P_{t}\geq\left( 1+\frac{\eta}{100} \right)\times min\left( P_{t-1},P_{t-2},\cdots,P_{t-k} \right) \\ 0,else \end{aligned} \right.$  $S_{t,FR}^{\mathrm{sell}}=\left\{ \begin{aligned} 1,P_{t}\leq\left( 1+\frac{\eta}{100} \right)\times max\left( P_{t-1},P_{t-2},\cdots,P_{t-k} \right) \\ 0,else \end{aligned} \right.$ |
| 112 | Technical transaction rules | MA | $S_{t,MA}=\left\{ \begin{aligned} 1,\mathrm{MA}_{s,t}\geq\mathrm{MA}_{l,t} \\ 0,else \end{aligned} \right.$ |
| 113 | Technical transaction rules | OSLT | $S_{t,OSLT}^{\mathrm{buy}}=\left\{ \begin{aligned} 1,RSI\leq50+\eta\\ 0,else \end{aligned} \right.$  $S_{t,OSLT}^{\mathrm{sell}}=\left\{ \begin{aligned} 1,RSI\geq50+\eta\\ 0,else \end{aligned} \right.$ |
| 114 | Technical transaction rules | SR | $S_{t,SR}^{\mathrm{buy}}=\left\{ \begin{aligned} 1,P_{t}\geq\left( 1+\frac{\eta}{100} \right)\times max\left( P_{t-1},P_{t-2},\cdots,P_{t-k} \right) \\ 0,else \end{aligned} \right.$  $S_{t,SR}^{\mathrm{sell}}=\left\{ \begin{aligned} 1,P_{t}\leq\left( 1+\frac{\eta}{100} \right)\times min\left( P_{t-1},P_{t-2},\cdots,P_{t-k} \right) \\ 0,else \end{aligned} \right.$ |

Wherein $\mathrm{high}_{t}$ is the highest stock price in t days, $\mathrm{low}_{t}$ is the lowest stock price in t days.$\mathrm{MA}_{j,t}=\left( \frac{1}{i} \right)\sum_{i=0}^{j-1} P_{t-i}$ ($j=s,l$). $s,l,k\in\left[ 1,3,6,9,12 \right]$, $\eta\in\left[ 5,10 \right]$, $s<l$.
